# An Improved Frequency Domain Guided Thermal Imager Strips Removal Algorithm Based on LRSID

**DOI:** 10.3390/s22197348

**Published:** 2022-09-28

**Authors:** Junchen Li, Li Zhong, Zhuoyue Hu, Fansheng Chen

**Affiliations:** 1 Key Laboratory of Intelligent Infrared Perception, Shanghai Institute of Technical Physics, Chinese Academy of Sciences, 500 Yu Tian Road, Shanghai 200083, China; 2 University of Chinese Academy of Sciences, Beijing 100049, China; 3 CAS Key Laboratory of Infrared System Detection and Imaging Technology, Shanghai Institute of Technical Physics, Shanghai 200083, China; 4 International Research Center of Big Data for Sustainable Development Goals (CBAS), Beijing 100094, China

**Keywords:** SDGSAT-1, thermal infrared, fringe noise, frequency domain guidance, image processing

## Abstract

The thermal imaging image of the Sustainable Development Science Satellite (SDGSAT-1) is mainly used for high-resolution observations of the ground width, due to the influence of blind elements and non-uniformity of the detector, and the system is a pendulum sweep imaging mode, resulting in fringed noise in the image. In this paper, a Fringing algorithm based on LRSID (low-rank-based single-image decomposition) algorithm is proposed, which can effectively remove the lateral and vertical fringe noise of the thermal imager and maintain the detail and clarity of the image. First, pretreatment of the obvious light and dark stripes then, based on LLSID algorithm, the vertical direction pinstripes and horizontal stripes are processed; finally, the fringed frequency band of the original image is replaced in the frequency domain with the image frequency domain processed by the LRSID algorithm, and then the Fourier inverse transformation is performed to obtain the final image. Using the method proposed in this paper, the simulated and actual SDGSAT-1 thermal imaging camera remote sensing stripes images are removed, and the visual and quantitative indicators are compared with the processing results of other algorithms, and the results show that the proposed algorithm has the best performance to remove the stripes, which can effectively remove horizontal and vertical fringes at the same time, and retain the detail and clarity of the image.

## 1. Introduction

Infrared imaging system has been widely used in military and civil fields. For thermal infrared remote sensing imaging system, as the infrared detector has the influence of blind pixels or non-uniformity, and it is scanning imaging, it will lead to more fringe noise in the image, which will greatly affect the quality of the image. Therefore, it is necessary to study the corresponding the fringe algorithm for processing, in order to facilitate the subsequent remote sensing applications.

At present, the research on image of fringe removal algorithm is very extensive. According to the basic principle of the algorithm, it can be divided into filtering algorithm, low rank model algorithm and deep learning correlation algorithm. The principles and characteristics of specific correlation algorithms are summarized as follows:

(1) Filtering algorithm

The research of filtering algorithms is relatively early. Its basic principle is to design corresponding filters according to the characteristics of the stripes in the image in the spatial domain or frequency domain, filter the image in the spatial domain or frequency domain, and achieve the removal of stripe noise. In 2006, Liu et al. Used Fourier transform and adaptive filtering to remove fringes from Landsat-7 remote sensing images [1]. There are also much research on the fringe algorithm related to the remote sensing image of MODIS satellite. The algorithm based on the combination of moment matching and surface filtering [2,3], the interpolation filtering algorithm [4], and the filtering algorithm based on multiple regression [5] are effectively applied to the de fringe of MODIS Image. In addition, the newest studied one-dimensional guidance filter (GF) algorithm for infrared image de fringe has a good effect, and is a relatively representative spatial domain filtering algorithm [6]. Representative frequency domain filtering methods include a-contrario algorithm based on statistics, which can effectively align periodic fringe noise for removal [7], and Fourier spectrum guidance algorithm (FSG) based on the characteristics of fringe in frequency domain, which also retains image details while removing fringe [8].

(2) Low rank model algorithm

Low rank model algorithm is a relatively new algorithm to remove fringes, which is mainly to solve the problem of multispectral coherence loss of remote sensing images. According to its principle, it can be divided into low rank matrix recovery model and low rank tensor recovery model. For the low rank matrix restoration model algorithm, the algorithms for hyperspectral image processing include orthogonal subspace learning algorithm [9], low rank matrix restoration [10], and total variational algorithm based on low rank representation [11]. General algorithms include low rank image decomposition (LRSID) [12] and transfer low rank matrix restoration algorithm [13]. For the low rank tensor recovery model algorithm, it is mainly to solve the damage of the low rank matrix recovery algorithm to the spectral spatial three-dimensional matrix structure. The more representative algorithms include the intrinsic tensor sparsity algorithm [14], the combination algorithm of SSTV and low rank tensor recovery [15,16], and the combination algorithm of SSTV and low rank tensor decomposition [17].

(3) Deep learning correlation algorithm

Deep learning algorithm also has a good effect on image of fringe removal, which has been widely used in recent years. This kind of algorithm usually does not need to analyze the spatial domain or frequency domain features of stripes, but only needs to train a large number of labeled data to get a better feature model, which can achieve efficient image of stripes removal. Among them, the deep learning removing fringes algorithm based on CNN convolution neural network has been widely used [18,19,20]. At present, a network with good performance is the two-step deep convolution neural network model based on wavelet [21], which can model fringes and images at the same time, and wavelet transform is used to extract multi-scale information.

At present, several kinds of the fringe removal algorithms have been studied more and have good results in certain application scenarios. However, for the thermal infrared image of the thermal imager of our sustainable development science satellite (SDGSAT-1), there will be some noise residue if we only use the filtering algorithm to process it because of the many kinds of stripes. Only using low rank model algorithm, the fringe removal effect is better, but the loss of detail information of the image is large. Although deep learning algorithms have good results in other applications, for SDGSAT-1 satellite, due to the lack of data sets, complex training models and time-consuming training, there are problems of low efficiency. Therefore, based on the characteristics of fringes in the frequency domain, combined with the low rank LRSID algorithm, this paper proposes an improved frequency domain guided strips removal algorithm: replace the fringe band of the original image in the frequency domain with the image processed by LRSID algorithm in the frequency domain, and then perform inverse Fourier transform to obtain the final image. It has achieved a very good effect in the thermal infrared image of the sustainable development science satellite (SDGSAT-1). The algorithm proposed in this paper is consistent with LRSID algorithm and frequency domain algorithm in computational complexity. The time complexity of our algorithm is O(n).

## 2. Materials and Methods

**Figure 1:**
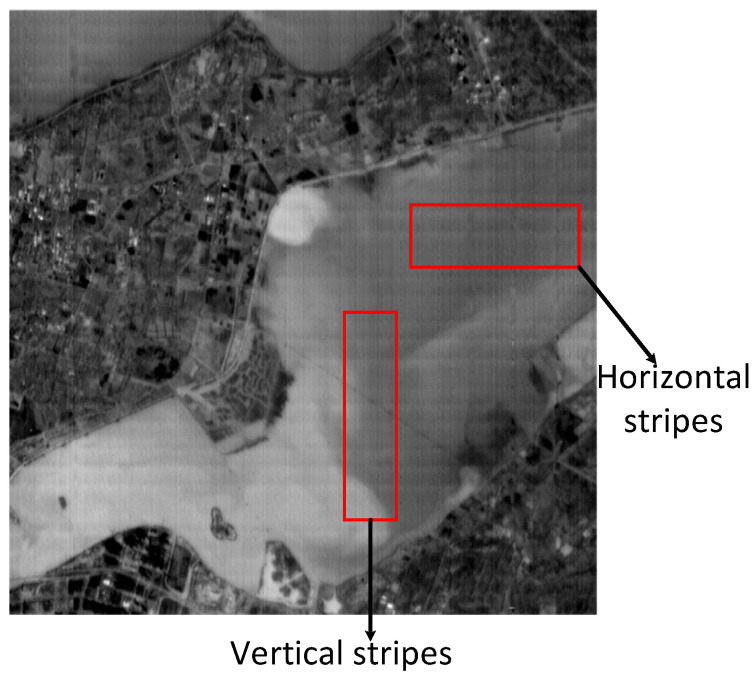
Original image from SDGSAT-1 (512 * 512).

Figure 1 shows the original image taken by the thermal imager of the sustainable development science satellite (SDGSAT-1) to the ground. From the figure, we can see that there are obvious bright and dark stripes, vertical fine stripes and horizontal periodic stripes in the image. According to its characteristics, this paper first preprocesses the obvious bright and dark fringes, and then processes the fine fringes and transverse fringes based on the algorithm combining LRSID and frequency domain guidance. The specific algorithm is introduced as follows. The size of the original image to be processed is 512 * 512 pixels.

(1) Preprocessing algorithm of obvious bright stripes

The obvious bright stripes and dark stripes are vertical stripes, which can be removed by pretreatment first. According to its characteristics, it can be preprocessed through the idea of threshold and replacement. Traverse the original image by column, calculate the average value of the current column and the two columns before and after, and then subtract the average value of the current column from the average value of the two adjacent columns respectively. If the difference is greater than the threshold value of 1, it indicates that the current column is an obvious bright stripe. Then, subtract the average value of the current column from the average value of the adjacent two columns. If the difference is greater than the threshold value of 2, it indicates that the current column is an obvious dark stripe. Both of the two threshold values are empirical which are selected according to actual stripe pattern type [8]. Save all the judged obvious bright and dark stripes, and finally perform the replacement operation.

The basic idea is to traverse the saved obvious stripe column, and first judge whether the latter column is a stripe column. If so, the current column is replaced by the previous column; otherwise, judge whether the previous column is a stripe column. If so, replace the current column with the latter column. If the above conditions are not met, and the current fringe column is an isolated column, replace it with the mean value of the previous and subsequent columns. The basic process of the obvious bright and dark fringe preprocessing Algorithm 1 is as follows:
**Algorithm 1:** preprocessing obviously bright and dark fringeInput: SDGSAT-1’s thermal infrared raw image ***raw_img***1: Set the threshold parameters ***th1, th2***2: Set the vector parameter ***S*** of obvious vertical stripe3: Set the count parameter ***ct = 0***4: Calculate the column number ***N*** of the ***raw_img***5: **for *i = 2: N − 1***6:    Calculate the mean value ***X(i − 1)*** of the column ***i − 1***7:    Calculate the mean value ***X(i)*** of the column ***i***8:    Calculate the mean value ***X(i + 1)*** of the column ***i + 1***9:    **if *X(i)* − *X(I − 1)*** > ***th1*** and ***X(i) − X(i + 1) > th1***10:      ***ct = ct + 1, S(ct) = i***11:   **if *X(i − 1) − X(i) > th1*** and ***X(i + 1) − X(i) > th1***12:      ***ct = ct + 1, S(ct) = i***13:   **end**14: Set the preprocessed image ***pre_img = raw_img***15: **for *j = 1: ct***16:    **if *S(j) + 1*** is belong to ***S***17:     ***pre_img(:, S(j)) = pre_img(:, S(j) − 1)***18:    **elseif *S(j) − 1*** is belong to ***S***19:     ***pre_img(:, S(j)) = pre_img(:, S(j) + 1)***20:    **else**21:     ***pre_img(:, S(j)) = pre_img(:, S(j) + 1)***22: **end**Output: SDGSAT-1’s thermal infrared preprocessing image ***pre_img***

(2) LRSID + frequency domain guidance

According to the characteristics of vertical fine fringes and horizontal periodic fringes after thermal infrared image preprocessing of the sustainable development science satellite (SDGSAT-1), this paper proposes an improved Fourier spectrum guided algorithm based on LRSID algorithm to remove fringes. LRSID algorithm is a fringe removal algorithm based on the concept of low rank single image decomposition. By considering the structural characteristics of the fringe itself, the de fringe problem is transformed into an image decomposition problem, which can effectively remove the vertical and horizontal fringes at the same time. For the specific principle and process of the algorithm, refer to [12].

Fourier spectrum guidance algorithm (FSG) is an algorithm that performs removing fringe processing in the frequency domain. By replacing and correcting the frequency band damaged by fringe noise, it can remove image fringes while ensuring image details. For the specific principle and process of the algorithm refer to [8].

Although the above two algorithms can process the image fringes to a certain extent, there are some problems in the removal of fringes in the thermal infrared image of SDGSAT-1. The LRSID algorithm can effectively remove the vertical fine fringes and horizontal fringes, but it will cause serious loss of image details. The frequency domain guidance algorithm can better maintain the details of the image while removing the fringe noise, However, the removal effect of vertical fine stripes and horizontal stripes is not obvious. Therefore, combining the characteristics of the two algorithms, this paper proposes an improved frequency domain guided fringe removal Algorithm 2 based on LRSID.
**Algorithm 2:** Striping algorithm based on LRSID and frequency domain guidanceInput: SDGSAT-1’s thermal infrared preprocessing image ***pre_img***1: Set the parameters ***opts*** of LRSID (the lagranian parameters, the regularization parameters and the number of iterations)2: Set the quantization bits ***Q*** of image3: Normalize the image ***pre_img/2^Q^***4: Implement the LRSID algorithm, obtain the initial destripe image ***destripe_img1***5: Restore to the raw scale destripe_img1 = destripe_img1*2^Q^6: Set the width of the stripe frequency band ***w1*** and ***w2***7: Calculate the Fourier Spectrum ***F1*** of ***pre_img***8: Calculate the Fourier Spectrum ***F2*** of ***destripe_img1***9: Calculate the row ***M*** and column ***N*** of the Fourier Spectrum,10: Calculate the center row of the Fourier Spectrum ***c1 = M/2 + 1***,11: Calculate the center column of the Fourier Spectrum ***c2 = N/2 + 1***12: Calculate the frequency band of vertical stripes ***SFB1 = [c1 − w1: c1 + w1, 1: N]***13: Calculate the frequency band of horizontal stripes ***SFB2 = [1: M, c2 − w2: c2 + w2]***14: The stripe frequency band of ***F1*** is replaced by ***F2***,***F3 = F1, F3(SFB1) = F2(SFB1), F3(SFB2) = F2(SFB2)***15: Calculate the Fourier inversion of the ***F3,*** and obtain the final stripe removed image ***destipe_img***Output: SDGSAT-1’s thermal infrared striping image ***destripe_img***

**Figure 2:**
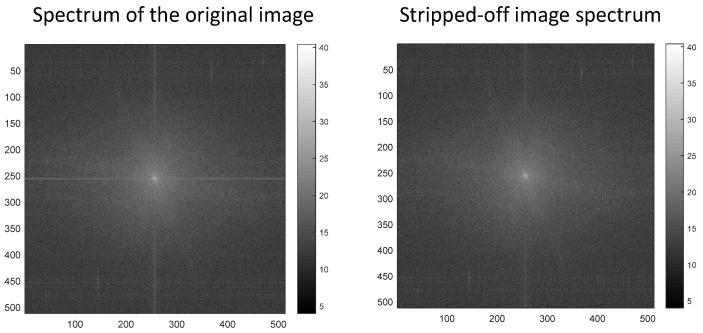
Spectrum of thermal infrared image from SDGSAT-1.

The basic idea is to use LRSID algorithm to process the preprocessed thermal infrared image, then calculate its spectrum, and calculate the spectrum of the preprocessed thermal infrared image at the same time. Then, the fringe band is replaced. According to the characteristics of the image fringe in the frequency domain, the bright line in the vertical direction of the spectrum center represents the horizontal fringe in the spatial domain, and the bright line in the horizontal direction of the spectrum center represents the vertical fringe in the spatial domain, which are all defined as the fringe band. The traditional frequency domain guidance algorithm uses the central frequency spectrum of the image after Gaussian filtering to replace the fringe frequency band. In this paper, the frequency spectrum of the image processed by LRSID algorithm is used to replace the fringe frequency band, and the radius of the central frequency band is selected as 2. Finally, the frequency spectrum after band replacement can be obtained by inverse Fourier transform. The specific algorithm flow is as follows. After implementing the algorithm, it can be seen from Figure 2 that the bright line of the fringe band at the center of the original spectrum is replaced, indicating that the fringes in the spatial domain have been effectively removed.

In order to verify the effectiveness of the proposed algorithm, the simulated thermal infrared remote sensing image and the original thermal imager image with noise are processed and analyzed respectively. The simulated image adopts L2 level non stripe noise data taken by landsat8 thermal infrared instrument [22] and is processed by adding stripe noise in the horizontal and vertical directions. The actual image adopts the level 0 data of our sustainable development science satellite (SDGSAT-1), with the influence of fringe noise. In addition, we use GF algorithm [6], a-contrario algorithm [7], LRSID algorithm [12], FSG algorithm [8], gf and FSG combination algorithm, a-contrario and FSG combination algorithm to process the image and compare the results. The experimental platform is a personal computer, the CPU is Intel Core i5 with 2.5 GHz, and the memory RAM size is 8 GB. Use MATLAB to realize the code of the algorithm.

## 3. Results

### 3.1. Simulation Image Data Processing and Result Analysis

**Figure 3:**
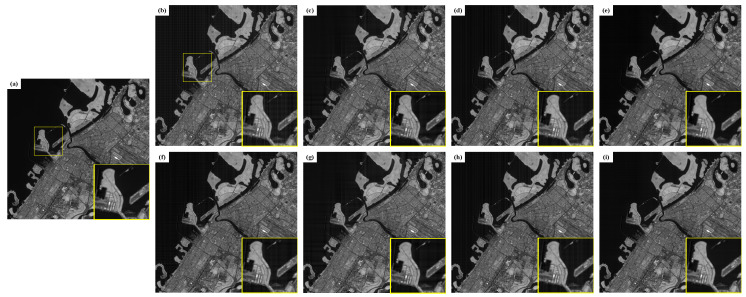
Comparison of simulation image processing results: (**a**) noiseless, (**b**) add fringe noise, (**c**) GF, (**d**) A-Contrario, (**e**) LRSID, (**f**) FSG, (**g** )GF + FSG, (**h**) A-Contrario + FSG, (**i**) Ours.

Figure 3 is a comparison of the results of different algorithms for deinterlacing the simulated image. The L2 level image of LandSat8 without fringe noise is shown in Figure 3a. The image with simulated fringe noise is shown in Figure 3b, which is simulated according to the fringe noise characteristics of the thermal infrared image of the sustainable development science satellite (SDGSAT-1), including vertical fine stripes and horizontal stripes. 

According to the simulation image processing results, the fringe removal algorithm based on GF can remove most of the fringes, but there is a certain amount of residual fringe noise. The simulation image results are as follows:

(1) The results of the combined processing based on GF and FSG frequency domain guidance still have corresponding problems, as shown in Figure 3c,g, respectively.
(2) The algorithm based on a-contrario can effectively remove horizontal stripes, but the effect of removing fine stripes in the vertical direction is poor.
(3) The algorithm based on the combination of a-contrario and FSG frequency domain guidance also has corresponding problems, as shown in Figure 3d and h, respectively.
(4) The results of processing based on a separate FSG frequency domain guidance algorithm is shown in Figure 3f. It can be seen that the algorithm has a certain effect on the removal of vertical fine stripes and horizontal stripes, but there are still many fringe noise residues. The main reason is that the frequency band of Gaussian filtering is used in the algorithm to replace the fringe band, and simple Gaussian filtering cannot completely remove the image fringe effectively.
(5) The results of processing based on LRSID algorithm are shown in Figure 3e. It can be seen that the vertical fine stripes and horizontal stripes have been effectively removed, but the image details are blurred.
(6) The processing results of the algorithm based on the combination of LRSID and frequency domain guidance proposed in this paper are shown in Figure 3i. It can be seen that the fringes have been effectively removed, and the image details are still preserved, which has the best de fringes effect compared with other algorithms.

**Figure 4:**
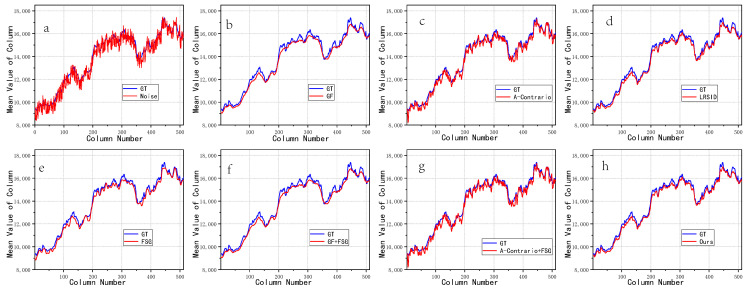
The variation of pixel mean value in the column direction of the simulation image: (**a**) add fringe noise, (**b**) GF, (**c**) A-Contrario, (**d**) LRSID, (**e**) FSG, (**f**) GF + FSG, (**g**) A-Contrario + FSG, (**h**) Ours.

Figure 4 shows the comparison between the change of the column pixel mean value of the simulated noise image and the de striped image and the change of the column pixel mean value of the truth (GT: ground truth) image. It can be seen from Figure 4a that due to the influence of fringe noise, the change curve of simulated noise image is quite different from that of GT image.

(1) The change curve of the striped image based on the a-contrario algorithm and the algorithm based on the combination of a-contrario and FSG frequency domain guidance is also far from that of the GT image, indicating that its de striping effect is poor.
(2) The change curve of the striped image based on GT algorithm and the algorithm based on the combination of GF and FSG frequency domain guidance is closer to the GT image curve, but smoother.
(3) The curve of the processed image based on FSG frequency domain guidance algorithm is also closer to the GT image, and the algorithm also has a certain effect.
(4) Based on LRSID algorithm and the algorithm proposed in this paper, the average change curve of column pixels in the removing striped image is the closest to that of GT image, which shows that the algorithm has the best strips removal effect.

In order to quantitatively evaluate the effect of the algorithm, the peak signal-to-noise (*PSNR*) and structure similarity (*SSIM*) [23] indexes of the simulated image and the de striped image are calculated, as shown in Table 1. It can be seen that the algorithm proposed in this paper has the highest *PSNR* and *SSIM*, and has the best de fringe effect.

(1) $$PSNR=10\times log_{10}\left(\frac{{\left(2^{n}-1\right)}^{2}}{MSE}\right)$$

*MSE* means the mean square error between original image and processed image.

(2) $$SSIM\left(x,y\right)=\frac{\left(2\mu_{x}\mu_{y}+c_{1}\right)\left(2\sigma_{xy}+c_{2}\right)}{\left(\mu_{x}^{2}+\mu_{y}^{2}+c_{1}\right)\left(\sigma_{x}^{2}+\sigma_{y}^{2}+c_{2}\right)}$$

$\mu_{x}$ is the mean value of *x*; $\mu_{y}$ is the mean value of *y*; $\sigma_{x}^{2}$ is the variance of *x*; $\sigma_{y}^{2}$ is the variance of *y*; $\sigma_{xy}$ is the covariance of *x* and *y*; $c_{1}$ and $c_{2}$ are constants.

At the same time, in order to quantitatively evaluate the changes in image details, the sharpness indexes of the truth (GT: ground truth) image and the image after de striping are calculated, as shown in Table 2. It can be seen that the clarity index of the true value image without fringe noise is the best. 

For the striped image, the image processed based on a-contrario algorithm and the algorithm based on the combination of a-contrario and FSG frequency domain guidance has the best sharpness, but the effect of de striping is poor. Compared with the LRSID algorithm, which has a better effect of removing fringes, the algorithm proposed in this paper can greatly improve the image definition, and can achieve a better effect of removing fringes while retaining the details of the image.

The evaluation indicators used in this paper are as follows [24]:

(1) Gray variance.

The image is the clearest, and the high-frequency components in the image are also the largest. The algorithm takes the average gray level of all pixels in the image as a reference, calculates the difference and squares the gray level of each pixel, and then normalizes it with the total number of pixels. It represents the average degree of gray level changes in the image.

(3) $$\bar{g}=\frac{1}{N_{x}\times N_{y}}\overset{N_{x}}{\underset{x=1}{\sum }}\overset{N_{y}}{\underset{y=1}{\sum }}f\left(x,y\right)$$

(4) $$s=\frac{1}{N_{x}\times N_{y}}\overset{N_{x}}{\underset{x=1}{\sum }}\overset{N_{y}}{\underset{y=1}{\sum }}{\left(f\left(x,y\right)-\bar{g}\right)}^{2}$$

(2) Absolute value of gray difference.

The sum of the absolute values of the image differences in the x direction and the y direction is used as the measurement standard:

(5) $$s=\overset{N_{x}-1}{\underset{x=1}{\sum }}\overset{N_{y}-1}{\underset{y=1}{\sum }}\left\{\left|f\left(x+1,y\right)-f\left(x,y\right)\right|+\left|f\left(x,y+1\right)-f\left(x,y\right)\right|\right\}$$

(3) Gary difference sum of squares.

The sum of the difference square values of the *x*-direction and y-direction images is used as the measurement standard to highlight the influence of the differential value and improve the signal-to-noise ratio:

(6) $$s=\overset{N_{x}-1}{\underset{x=1}{\sum }}\overset{N_{y}-1}{\underset{y=1}{\sum }}\left\{{\left(f\left(x+1,y\right)-f\left(x,y\right)\right)}^{2}+{\left(f\left(x,y+1\right)-f\left(x,y\right)\right)}^{2}\right\}$$

(4) Brenner function.

Calculate the square of the gray difference between two adjacent pixels:

(7) $$s=\overset{N_{x}-2}{\underset{x=1}{\sum }}\overset{N_{y}-2}{\underset{y=1}{\sum }}{\left(f\left(x+2,y\right)-f\left(x,y\right)\right)}^{2}$$

(5) Roberts gradient sum.

Sum of absolute values of gray value differences of diagonal pixels of adjacent four pixels:

(8) $$s=\overset{N_{x}-1}{\underset{x=1}{\sum }}\overset{N_{y}-1}{\underset{y=1}{\sum }}\left\{\left|f\left(x,y\right)-f\left(x+1,y+1\right)\right|+\left|f\left(x+1,y\right)-f\left(x,y+1\right)\right|\right\}$$

(6) Laplace gradient sum 1.

Obtain the Laplacian gradient value of the pixel using the Laplacian template, and find the sum of the Laplacian gradient values of all pixels.

Laplace template:

(9) $$s=\overset{N_{x}-1}{\underset{x=2}{\sum }}\overset{N_{y}-1}{\underset{y=2}{\sum }}\left|f\left(x+1,y\right)+f\left(x-1,y\right)+f\left(x,y+1\right)+f\left(x,y-1\right)-4f\left(x,y\right)\right|$$

(10) $$m=\left[\begin{array}{ccc} 0 & 1 & 0\\ 1 & -4 & 1\\ 0 & 1 & 0 \end{array}\right]$$

(7) Laplace gradient sum 2.

Obtain the Laplacian gradient value of the pixel using the Laplacian template, and find the sum of the Laplacian gradient values of all pixels.

Laplace template:

(11) $$\begin{array}{ll} \nabla f\left(x,y\right)=f\left(x,y\right) & *m\\ & =[f\left(x+1,y\right)+f\left(x-1,y\right)+f\left(x,y+1\right)+f\left(x,y-1\right)+f(x\\ & -1,y-1)+f\left(x-1,y+1\right)+f\left(x+1,y-1\right)+f(x+1,y\\ & +1)-8f\left(x,y\right)] \end{array}$$

(12) $$s=\overset{N_{x}-1}{\underset{x=2}{\sum }}\overset{N_{y}-1}{\underset{y=2}{\sum }}\left|\nabla f\left(x,y\right)\right|$$

(13) $$m=\left[\begin{array}{ccc} 1 & 1 & 1\\ 1 & -8 & 1\\ 1 & 1 & 1 \end{array}\right]$$

(8) Tenengrad function.

The gradient function uses Sobel operator to extract the gradient values in the horizontal and vertical directions respectively.

Template operator:

(14) $$K_{x}=\left[\begin{array}{ccc} 0 & -1 & 0\\ 0 & 2 & 0\\ 0 & -1 & 0 \end{array}\right],\;K_{y}=\left[\begin{array}{ccc} 0 & 0 & 0\\ -1 & 2 & -1\\ 0 & 0 & 0 \end{array}\right]$$

Convolution result:

(15) $$f_{x}\left(x,y\right)=f\left(x,y\right)*K_{x}\;,\;f_{y}\left(x,y\right)=f\left(x,y\right)*K_{y}$$

Ambiguity value:

(16) $$s=\sqrt{f_{x}^{2}\left(x,y\right)+f_{y}^{2}\left(x,y\right)}$$

(9) Frequency domain evaluation.

The two-dimensional Fourier transform coefficient of the image represents the size of each frequency component of the image, so the modulus of the Fourier transform coefficient is used as the blur evaluation value:

(17) $$F\left(u,v\right)=\overset{N_{x}}{\underset{x=1}{\sum }}\overset{N_{y}}{\underset{y=1}{\sum }}f\left(x,y\right)e^{\left[-j2\pi \left(xu/N_{x}+yv/N_{y}\right)\right]}$$

(18) $$s={\left[Re\left(\frac{F\left(u,v\right)}{\sum_{x=1}^{N_{x}}\sum_{y=1}^{N_{y}}f\left(x,y\right)}\right)\right]}^{2}+{\left[Im\left(\frac{F\left(u,v\right)}{\sum_{x=1}^{N_{x}}\sum_{y=1}^{N_{y}}f\left(x,y\right)}\right)\right]}^{2}$$

(10) Vollaths function.

(19) $$s=\underset{y}{\sum }\underset{x}{\sum }f\left(x,y\right)*f\left(x+1,y\right)-M*N*\mu^{2}$$

### 3.2. Real Image Processing and Result Analysis of SDGSAT-1 Thermal Imager

**Figure 5:**
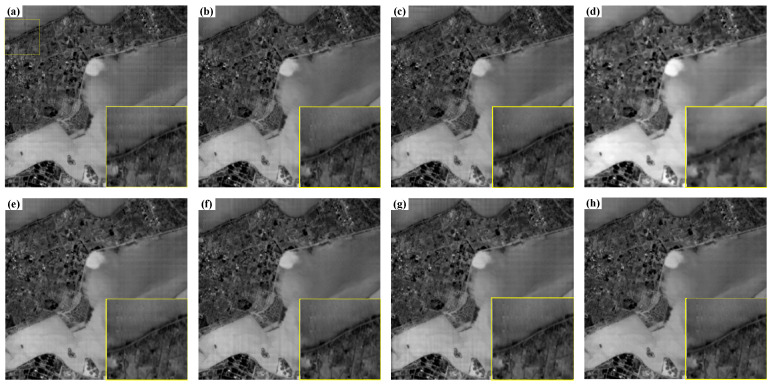
Comparison of actual image processing results of thermal imager: (**a**) origin. (**b**) GF. (**c**) A-Contrario. (**d**) LRSID. (**e**) FSG. (**f**) GF + FSG. (**g**) A-Contrario + FSG. (**h**) Ours.

The real image of SDGSAT-1 thermal imager and the image results after de striping are shown in Figure 5. It can be seen that compared with the original thermal infrared images, the stripes removal image has a certain effect. After the image is processed based on GF algorithm and the combination of GF and FSG frequency domain guidance, there are certain fringe noise residues at the edge of the image and in the region with large response. The images processed based on a-contrario algorithm and the combination of a-contrario and FSG frequency domain guidance algorithm still have obvious vertical stripes, and there are also many horizontal stripes left. Based on a separate FSG frequency domain guidance algorithm, there is also more fringe noise. The image processed based on LRSID algorithm can effectively remove the vertical fine stripes, but the details of the image are also blurred. The algorithm proposed in this paper has the best effect of removing fringes, while preserving the details of the image.

For the real fringe image of the thermal imager, there is no GT truth value image for comparison, so it is impossible to use the two indicators of *PSNR* and *SSIM* in the simulated image for evaluation. In order to quantitatively evaluate the effect of the real image de fringe algorithm, this paper calculates the inverse coefficient of variation (*ICV*) and mean relative deviation (*MRD*) of the image, where *ICV* is to intercept uniform image blocks for calculation, and *MRD* is to intercept image blocks with edges for calculation. The specific calculation results are shown in Table 3. 

*ICV* means the reciprocal of coefficient of variation:

(20) $$ICV=\frac{\mu }{\sigma }$$

*MRD* means the mean deviation divided by the mean value:

(21) $$MRD=\frac{\bar{d}}{\bar{x}}\times 100\%$$

It can be seen that the highest *ICV* value is obtained based on the processing result of LRSID algorithm. This is because the algorithm has a large image smoothing process, so the smoothness of the uniform area of the image is the best. However, due to the large smoothing of the algorithm, more image detail information is lost. In addition to the LRSID algorithm, the algorithm proposed in this paper can get the highest *ICV* value, and the *MRD* value obtained by the algorithm proposed in this paper is the smallest, indicating that the difference from the original image is the smallest, which can effectively remove the fringes and better retain the original information. 

Similarly, in order to quantitatively evaluate the changes in image details, calculate the sharpness index of the image after removing strips, as shown in Table 4. It can be seen that the sharpness index of the image based on LRSID algorithm is the lowest, and the sharpness of the image is the worst. The sharpness indexes of other strips removal algorithms are relatively close. Among them, the algorithm based on the combination of a-contrario and FSG frequency domain guidance and the algorithm proposed in this paper have the best sharpness. 

According to the article [24], Histogram Concentration (HC) can be used as an index to evaluate image clarity. It specifically shows that the more blurred the image is, the more the histogram is concentrated near the mean value. Therefore, we can use the probability of occurrence of the gray value near the average gray value of the image on the histogram to characterize the degree of blur of the image. The results are presented in Table 3.

According to the analysis of quantitative evaluation results, it can be found that the algorithm proposed in this paper has the best performance.

## 4. Discussion

In order to effectively remove the fringes from the image of the thermal imager of the sustainable development science satellite (SDGSAT-1), an improved frequency domain guided de fringes algorithm based on LRSID is proposed in this paper. The image spectrum processed by LRSID algorithm is used to replace the fringe noise band, which realizes the effective De Fringes Processing and retains the image details.

At the same time, GF algorithm, a-contrario algorithm, LRSID algorithm, FSG algorithm, gf and FSG combined algorithm, a-contrario and FSG combined algorithm, and the algorithm proposed in this paper are respectively used to process and compare the simulated thermal infrared remote sensing image and the original thermal image with noise. The results show that the algorithm proposed in this paper can achieve the best visual effect of removing stripes for both the simulation and the actual image, and the quantitative evaluation index is also the best. Since the threshold setting of the algorithm is a priori, the versatility of the algorithm needs to be improved. The algorithm still needs higher performance hardware support in processing higher resolution remote sensing images, otherwise the processing efficiency will be reduced.

## Figures and Tables

**Table 1:** Comparison of quantitative indexes of simulation image processing results.

Index	Noisy	GF	A-Contrario	LRSID	FSG	GF + FSG	A − Contrario + FSG	Ours
PSNR	42.11	44.15	45.82	44.13	45.58	44.18	45.90	48.66
SSIM	0.67	0.82	0.73	0.72	0.75	0.79	0.73	0.86

**Table 2:** Contrast of sharpness index of simulation image after striping removal.

Index	GT	GF	A-Contrario	LRSID	FSG	GF + FSG	A − Contrario + FSG	Ours
Gray variance	1.77 × 10^7^	1.70 × 10^7^	1.77 × 10^7^	1.71 × 10^7^	1.75 × 10^7^	1.72 × 10^7^	1.76 × 10^7^	1.77 × 10^7^
Absolute value of gray difference	3.78 × 10^8^	3.80 × 10^8^	3.91 × 10^8^	2.62 × 10^8^	3.86 × 10^8^	3.82 × 10^8^	3.78 × 10^8^	3.92 × 10^8^
Gray difference sum of squares	8.13 × 10^11^	7.92 × 10^11^	8.13 × 10^11^	5.47 × 10^11^	7.99 × 10^11^	7.98 × 10^11^	8.09 × 10^11^	8.17 × 10^11^
Brenner function	1.00 × 10^12^	9.68 × 10^11^	1.01 × 10^12^	7.42 × 10^11^	9.84 × 10^11^	9.78 × 10^11^	9.97 × 10^11^	1.01 × 10^12^
Roberts gradient sum	4.73 × 10^8^	4.75 × 10^8^	4.96 × 10^8^	3.61 × 10^8^	4.84 × 10^8^	4.79 × 10^8^	4.74 × 10^8^	4.98 × 10^8^
Laplace gradient sum 1	4.36 × 10^8^	4.37 × 10^8^	4.47 × 10^8^	3.04 × 10^8^	4.41 × 10^8^	4.38 × 10^8^	4.37 × 10^8^	4.48 × 10^8^
Laplace gradient sum 2	1.05 × 10^9^	1.05 × 10^9^	1.08 × 10^9^	7.57 × 10^8^	1.06 × 10^9^	1.05 × 10^9^	1.05 × 10^9^	1.08 × 10^9^
Tenengrad function	1.58 × 10^9^	1.58 × 10^9^	1.65 × 10^9^	1.32 × 10^9^	1.62 × 10^9^	1.60 × 10^9^	1.58 × 10^9^	1.66 × 10^9^
Frequency domain evaluation	3.12 × 10^5^	3.09 × 10^5^	3.11 × 10^5^	2.43 × 10^5^	3.09 × 10^5^	3.10 × 10^5^	3.11 × 10^5^	3.12 × 10^5^
Vollaths function	3.07 × 10^12^	3.82 × 10^12^	3.02 × 10^12^	3.14 × 10^12^	2.12 × 10^12^	3.06 × 10^12^	3.04 × 10^12^	3.15 × 10^12^

**Table 3:** Comparison of quantization index of actual image processing results of thermal imager.

Index	Origin	GF	A-Contrario	LRSID	FSG	GF + FSG	A − Contrario + FSG	Ours
ICV	1017.23	1114.13	905.04	1748.65	1130.02	1101.57	821.14	1253.43
MRD	0.00%	0.14%	0.21%	0.13%	0.10%	0.13%	0.15%	0.07%
HC	0.26	0.15	0.13	0.08	0.12	0.10	0.06	0.05

**Table 4:** Contrast of sharpness index of actual image of thermal imager after stripe removal.

Index	GF	A-Contrario	LRSID	FSG	GF + FSG	A − Contrario + FSG	Ours
Gray variance	520	526	499	531	522	532	531
Absolute value of gray difference	1.66 × 10^6^	1.64 × 10^6^	7.20 × 10^5^	1.67 × 10^6^	1.67 × 10^6^	1.68 × 10^6^	1.68 × 10^6^
Gray difference sum of squares	1.20 × 10^7^	1.15 × 10^7^	3.32 × 10^6^	1.22 × 10^7^	1.21 × 10^7^	1.23 × 10^7^	1.23 × 10^7^
Brenner function	1.52 × 10^7^	1.42 × 10^7^	5.55 × 10^6^	1.55 × 10^7^	1.54 × 10^7^	1.56 × 10^7^	1.56 × 10^7^
Roberts gradient sum	2.08 × 10^6^	2.04 × 10^6^	1.06 × 10^6^	2.10 × 10^6^	2.09 × 10^6^	2.12 × 10^6^	2.11 × 10^6^
Laplace gradient sum 1	1.92 × 10^6^	1.89 × 10^6^	4.69 × 10^5^	1.92 × 10^6^	1.92 × 10^6^	1.93 × 10^6^	1.93 × 10^6^
Laplace gradient sum 2	4.35 × 10^6^	4.24 × 10^6^	1.27 × 10^6^	4.37 × 10^6^	4.36 × 10^6^	4.38 × 10^6^	4.37 × 10^6^
Tenengrad function	6.73 × 10^6^	6.66 × 10^6^	4.32 × 10^6^	6.86 × 10^6^	6.79 × 10^6^	6.92 × 10^6^	6.85 × 10^6^
Frequency domain evaluation	1.19 × 10^3^	1.17 × 10^3^	4.01 × 10^3^	1.20 × 10^3^	1.20 × 10^3^	1.20 × 10^3^	1.20 × 10^3^
Vollaths function	2.00 × 10^9^	1.95 × 10^9^	7.68 × 10^9^	2.02 × 10^9^	2.01 × 10^9^	2.02 × 10^9^	2.01 × 10^9^

## Data Availability

Not applicable.
